# The Role of the Face Itself in the Face Effect: Sensitivity, Expressiveness, and Anticipated Feedback in Individual Compliance

**DOI:** 10.3389/fpsyg.2018.02499

**Published:** 2019-01-04

**Authors:** Maggie Wenjing Liu, Qichao Zhu, Yige Yuan

**Affiliations:** School of Economics and Management, Tsinghua University, Beijing, China

**Keywords:** face effect, compliance, feedback, facial expression, facial expressiveness, interpersonal sensitivity

## Abstract

Face-to-face interactions are central to many individual choices and decision-making issues, such as customer services, sales, promotions, and negotiations. While the face effect, that is, face-to-face interactions are more effective in inducing compliance than other forms of interactions, has been noted in the literature, its mechanism has rarely been explored. This research helps to fill the theoretical void and provides new insights into the face effect with two lab experiments and one field experiment. Study 1, a field experiment conducted in a beauty salon, and Study 2, a lab experiment, show that the face effect is largely attributable to anticipated facial feedback and that the face effect is stronger when individuals are sensitive to face and when the requester’s face is expressive. Study 3, using video-simulated face-to-face interactions, demonstrates that anticipated facial feedback, not necessarily actual feedback, is enough to drive the face effect. In so doing, this research furthers our understanding of factors that affect individual compliance in face-to-face interactions in both the “sending” and “receiving” stages. We discuss the theoretical and empirical implications, limitations, and future avenues of research.

## Introduction

Face-to-face interactions are one of the most pervasive and important forms of interpersonal interactions (Kendon et al., 1975). Additionally, these interactions are central to many individual choices and decision-making issues, such as customer service, sales, promotions, and negotiations. Most critically, face-to-face interactions allow individuals to receive nonverbal cues that are absent or incomplete in other forms of interactions (e.g., telecommunications; Short et al., 1976; Song et al., 2016). In a broad sense of face-to-face communications, recent development of digital technologies has increased the extent of individuals interacting face to face. Users of both cellphones and computers have increasingly utilized their video calling functions to facilitate face-to-face interactions (Lin and Liu, 2009). For example, Apple’s iPhone video calling application, Facetime, is being used extensively around the world. Many companies (e.g., Verizon Wireless, IKEA, and Continental Airlines) have already added “virtual agents,” that is, online customer service representatives with human-like faces and responses, to their websites to facilitate decisions and choices (Wooters and Marcu, 2009). In addition, Emoji, a series of two-dimensional pictographs including facial expressions to produce emotions and facial feedback similar to those in face to-face interactions, has become popular in e-communications (Kelly and Watts, 2015). These advances suggest that despite technological developments that have changed the ways in which face-to-face interactions occur, such interactions remain critical for individual choices and decisions.

This research is motivated by the pervasive compliance problems in face-to-face interactions in both daily lives and marketing activities. Factors inducing consumer compliance have been an important area in the literature (e.g., Tybout, 1978; Mowen and Cialdini, 1980; Wosinska, 2005). Some examples of compliance problems include decisions related to buying a product, donating to a charity, or, generally, complying with a request. The literature in psychology, communications, and political science has noted the face effect, i.e., face-to-face interactions are more effective in inducing compliance than other forms of interactions, such as direct mail, telephone calls, and emails (e.g., Milgram, 1965b; Gerber and Green, 2000; Roghanizad and Bohns, 2017).

Facial expressions are mainly used to display emotions to others in social interactions (Ekman, 1993). The facial feedback, or information conveyed by facial expressions, is an essential factor used by individuals to infer others’ personalities and intents from their faces (Todorov et al., 2008; Ueda et al., 2017). On the other hand, the effect of facial feedback is not necessarily limited to actual facial expressions. Prior research has found that social influence can occur while others are not physically present, but simply anticipated to be present or imagined (Modigliani, 1971). Therefore, anticipated facial feedback can be a pivotal factor. Despite the pervasiveness of the face effect on compliance decisions, mechanism of the face effect has rarely been explicitly explored (Gerber and Green, 2000). To address this gap in theory, we propose and test anticipated facial feedback as one important underlying mechanism of the face effect with one field study and two lab experiments. We also examine the roles of facial expressiveness and sensitivity to face in amplifying or mitigating anticipated facial feedback, identifying two important boundary conditions of the face effect. Furthermore, we test the notion that anticipated facial feedback, rather than actual facial feedback, drives compliance with request during face-to-face interactions (Liu, 2010).

The rest of the article is organized as follows. First, we present a review of the literature related to the various conceptual elements of our research: face-to-face interactions, compliance, facial feedback, sensitivity to face, and facial expressiveness. Second, we develop the theoretical framework and propose multiple research hypotheses. Third, we report the procedures and results of two laboratory experiments and a field experiment to test the hypotheses. Finally, we conclude the article with the theoretical contributions, empirical implications, limitations, and future research directions.

## Literature Review and Hypothesis Development

### Effect of Face-to-Face Interactions on Compliance

Goffman (1959, p. 233) defines face-to-face interactions as “the reciprocal influence of individuals upon one another’s actions when in one another’s immediate physical presence.” However, with the development of technologies, such as video calls and the Internet, individuals no longer need to be physically present with their interactive partners. Thus, we define face-to-face interactions as personal communications in which individuals can see the face of their interactive partner.

In this article, we focus on individual decisions related to compliance, which represents one of the most common types of decisions during interpersonal interactions (Cialdini and Goldstein, 2004). Compliance refers to the act of responding favorably to a request made by another individual (i.e., the requester; Cialdini, 2001). A request can be explicit (e.g., a phone call soliciting donations) or implicit (e.g., an advertisement meant to induce purchase of products). Individuals often comply with requests when they are motivated to develop and preserve significant interpersonal relationships or maintain a favorable self-concept (Cialdini and Goldstein, 2004; Andrighetto et al., 2015). Accordingly, the principle of “social validation” describes an individual’s tendency to look into other individuals for cues regarding how to think, feel, and behave (Cialdini, 2001).

Classical compliance research indicates that different variables of face-to-face interactions, such as effort expended in the interactions (Zimbardo and Ebbesen, 1970) and behavior of experiment confederates (Milgram, 1965a), influence compliance. Marketing studies have examined compliance-inducing strategies, particularly in the personal selling and advertising areas (e.g., Tybout, 1978; Mowen and Cialdini, 1980; Wosinska, 2005). For instance, Wosinska (2005) suggests direct-to-consumer advertising of medicines as a good way of inducing compliance, since it empowers consumers and meets their demand for information.

More recent research has noted the face effect (e.g., Milgram, 1965b; Gerber and Green, 2000; Roghanizad and Bohns, 2017). For example, Gerber and Green (2000) found that personal canvassing increases voter turnout more than direct mail and telephone calls in a field experiment. Roghanizad and Bohns (2017) suggest that people often underestimate compliance rate in face-to-face interactions while overestimating compliance rate of emails due to varied trust and empathy levels of the two channels. Yet the underlying mechanism of the face effect has rarely been explicitly explored (Gerber and Green, 2000). As face-to-face interactions contain numerous nonverbal cues and feedbacks (Short et al., 1976; Song et al., 2016), and anticipated social disapproval drives social influence in an imagined face-to-face interaction (Modigliani, 1971), we next review the role of anticipated facial feedback in the face effect.

### Facial Feedback, Sensitivity, and Expressiveness

Social presence theory indicates that face-to-face interactions are a high social presence medium and contain numerous overt and hidden communication channels (Short et al., 1976). Common nonverbal channels include facial expressions, eye behavior, head movements, hand/arm movements, hair, and make-up (Knapp and Hall, 2005). The face, however, is the most distinctive feature of the human body and most capable of revealing an individual’s emotions (Fanghänel et al., 2006; Schwaninger et al., 2006). The face is the most communicative part of our emotions, thus making it the most expressive part and the main interactive side of our body (Widen and Russell, 2004; Fanghänel et al., 2006; Schwaninger et al., 2006). The successful nonverbal communication of affect includes “sending” and “receiving” nonverbal messages accurately (Buck et al., 1972; Buck et al., 1974). Yet people are not equally able to be judged by and judge others accurately in unfamiliar situations, and this ability can be affected by some individual variables, such as gender, personality traits, and nonverbal skills (Ambady et al., 1995). For instance, females are more effective in communicating effects than males, as female senders have higher facial expressiveness (Buck et al., 1972; Buck et al., 1974).

Facial expressiveness relates to the “sending” of nonverbal messages and represents one of the face’s most critical functions in relation to human interactions. Hobson (1993) claims that individuals directly demonstrate their affective states to others through increasingly sophisticated facial expressions. Facial expressiveness can also be varied so that people may show blank facial expressions even if they experience certain emotions (Ekman, 1993). In fact, “emotional suppression,” meaning reducing emotional expressiveness intentionally, often occurs in a state of emotional arousal (Gross and Levenson, 1993).

Sensitivity, or interpersonal sensitivity, on the other hand, relates to the “receiving” of messages and is defined as accuracy in judging, noticing, and recalling cues given by the expressers (Hall et al., 2005). An individual’s sensitivity to nonverbal cues is an important part of human psychosocial functioning and emotional intelligence (Hall et al., 2009). To illustrate, Bernieri (1991) demonstrated that individuals with higher sensitivity to nonverbal cues learn more in face-to-face interactions than in distant modes of communications. Buck et al. (1972) suggest that females’ heightened interpersonal sensitivity underlies their effectiveness in receiving nonverbal cues (see also Thompson and Voyer, 2014).

Since face is the main interactive side of our body (Widen and Russell, 2004), face-to-face interactions are likely to generate a feedback mechanism between individuals and their interactive partners. Charles Darwin’s work on expression of emotions, first published in 1872, suggests that the movements of expression reveal not only emotions but also “the thoughts and intentions of others more truly than do words, which may be falsified” (p. 359). Therefore, people pay attention to the facial expressions of their interactive partners and infer their personalities and intents from these expressions (Todorov et al., 2008). For example, smile is usually a sign of trustworthiness, while anger and disgust are usually signs of trait dominance (Ueda et al., 2017; Ueda and Yoshikawa, 2018). Subjective expected pleasure theory indicates that individuals make choices to maximize expected pleasure formed by weighing and combining their anticipated feelings for each option (Mellers et al., 1999; Mellers and McGraw, 2001). During face-to-face interactions, when presented with a request, individuals are aware that their decisions will be evaluated immediately, and the emotions (positive ones with a “yes” such as happiness and relief, and negative ones with a “no” such as anger and disappointment) are likely to manifest on the requester’s face (Ekman, 1993; Fanghänel et al., 2006; Schwaninger et al., 2006). Therefore, we propose that anticipated facial feedback from the requester is an important source of information in deciding whether to comply with requests and that the individual will try to promote positive facial feedbackand/or inhibit negative one from the requester. In fact, previous research has found that positive facial expressions such as happiness promote approaching behaviors of the observer, while negative facial expressions such as anger promote avoidant behaviors of the observer (Ruggiero et al., 2017).

Bohns (2016) suggests that awkwardness from saying no to requests drives many compliance decisions. In fact, social influence can occur even when others are anticipated to be present, not physically present, and imagined or anticipated social disapproval drives this effect (Modigliani, 1971). As an imagination of experience after responding favorably to the request, anticipated positive facial feedback may also enhance the likelihood of compliance (Petrova and Cialdini, 2008). Therefore, we propose that the face effect is largely attributable to the individual’s anticipated facial feedback from the requester.

**H1**: The face effect is mediated by anticipated facial feedback from the requester.

Interpersonal sensitivity is a critical factor in individual decision-making. For instance, individuals in a subordinate position generally have higher interpersonal sensitivity than their bosses, since low-status members’ resources are often determined by their high-status partners (Kenny et al., 2010). In the present research, we focus on sensitivity to face. When individuals are sensitive to the requester’s face, they are more likely to attend to and react to the facial feedback. Hall et al. (2005) pointed out that individuals tend to be in a general state of being consciously sensitive to cues from their interactive partner, but can be less sensitive when there is a distraction. On the other hand, exposure to a brief orientation related to facial movements increases sensitivity to emotions communicated by facial expressions (Solomon et al., 1997). When individuals are insensitive to the requester’s face, however, the face effect will disappear because of the failure of “receiving” anticipated facial feedback. Therefore, we propose that the individual’s sensitivity to face moderates the face effect.

**H2**: The face effect is stronger when individuals are sensitive to face and weaker when they are insensitive to face.

Although much of the literature on facial expressiveness has focused on its effect on others’ ability to interpret one’s emotions with facial expressions (e.g., Widen and Russell, 2004), expressiveness is also a basic part of individual ability to influence others in face-to-face interactions (Friedman et al., 1980). For example, a salesperson good at persuading consumers to buy something tends to be characterized by high nonverbal expressiveness, particularly in face-to-face persuasions (Friedman et al., 1980). Correlational studies have found that facial expressiveness is positively correlated with trait extraversion and negatively correlated with trait neuroticism (Riggio and Riggio, 2002), while extraversion is positively related to persuasiveness and neuroticism is negatively related to persuasiveness (Oreg and Sverdlik, 2014). We therefore hypothesize that the requester’s facial expressiveness will moderate the face effect. When the requester’s face is inexpressive, the face effect will disappear due to the failure of “sending” anticipated facial feedback.

**H3:** In face-to-face interactions, individuals are more likely to comply with a request when the requester’s face is expressive than when the requester’s face is inexpressive.

We next conduct one field study and two lab experiments to test our hypotheses.

## Study 1

We conducted a field study in a beauty salon to test the face effect in a real-world setting and H2 that the face effect is stronger when individuals are sensitive to face and weaker when they are insensitive. The beauty salon is located in an Asian city with a population of 2.8 million. The salon manager agreed to help with the experiment in exchange for advice on promotional strategies.

### Participants, Design, and Procedure

One hundred and twenty-six female customers participated in the experiment. The experiment used a 2 (promotion type: face-to-face request vs. written request) × 2 (sensitivity priming: sensitive to face vs. sensitive to hair) between-subjects design. Two specific services—hair cutting and facial cleaning—were used in this study, since they were both basic services and were offered at the same price in the salon. Solomon et al. (1997) showed that exposure to a brief orientation related to facial movements increases sensitivity to emotions communicated by facial expressions. Hair was employed in this experiment as a competing channel against face (Hall et al., 2005). During a hair cutting session, customers had their hair washed, cut, and blown dry and then received suggestions from stylists on how to style and care for their hair. During a facial cleaning session, customers received a deep cleaning of their face, a facial massage, plucking of the eyebrows and suggestions from stylists on skin care. Both services ranged from 20 to 30 min in duration. Customers who required both hair service and facial service in one trip were excluded from the experiment.

The customers entered the salon and required a certain type of service for themselves. Depending on the service they requested upon entering the store, customers self-selected themselves into either the sensitive to face group or the sensitive to hair group. Following the manipulation by Solomon et al. (1997), sensitivity (sensitive to face vs. sensitive to hair) was heightened by 1) stylists stimulating the customer’s face (vs. cutting the customer’s hair) for about half an hour and 2) stylists talking with the customer about taking care of the face (vs. hair) in the service session.

The salon offered a 20% discount on all services if customers purchased a membership card. The price of the membership card was equivalent to US$30. After the service, the stylist requested the customers to buy a membership card, either *via* face-to-face conversation or by giving the customer the membership card booklet (written request). The booklet described the promotion as customers getting 20% discount on all services offered by the salon when purchasing a VIP membership card. The customers could tick the “Yes” box on the booklet indicating their compliance if they chose to purchase the membership card. In the face-to-face conversations, the stylist described the same promotion for the VIP membership card and asked the customer directly whether they chose to buy it or not. Both the face-to-face conversation and the booklet reading occurred between the end of the service and payment for the service, so that the customers could benefit from the price discount instantly if they decided to purchase the membership card. As the dependent variable, if the customer chose to pay for the card at the end of the service, her choice was coded as compliance with promotion. Written informed consent was obtained from the participating customers in this study.

### Results and Discussion

**Figure 1:**
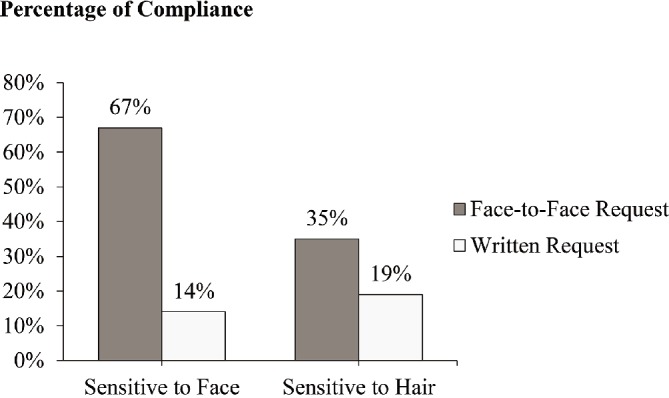
Compliance with promotion as a function of promotion type and sensitivity priming in Study 1.

We analyzed the data using a logistic regression model with compliance as the dependent variable, and promotion type and sensitivity priming as two independent variables. The results showed a nonsignificant simple effect of promotion type (*χ* ^2^(1) = 2.17 and *p* = 0.14) and a nonsignificant simple effect of sensitivity priming (*χ* ^2^(1) = 0.36 and *p* = 0.55), qualified by a significant interaction between promotion type and sensitivity priming (*χ* ^2^(1) = 3.84 and *p* = 0.05). As can be seen in Figure 1, the face effect was moderated by sensitivity to face. Customers receiving a facial service were more likely to purchase the membership card under the face-to-face promotion than under the written request (67 vs. 14%; *χ* ^2^(1) = 13.97 and *p* < 0.001). This difference between face-to-face promotion and written request conditions (35 vs. 19%; *χ* ^2^(1) = 2.17 and *p* = 0.14) was nonsignificant when customers received a hair service.

In conclusion, the beauty salon experiment demonstrated the face effect in a real-world setting. This study showed that face-to-face interactions increased likelihood of customer compliance with the request to buy a membership card, compared with situations without face-to-face interactions. Customer sensitivity to face moderated the face effect, such that the face effect was stronger when customers were sensitive to face and weaker when they were insensitive, consistent with H2. One weakness of Study 1 was that different sensitivity priming groups were divided using a self-selection method rather than randomization, due to the limitations of field experiments. Next, we conducted two lab studies to examine the hypotheses in a more controlled setting.

## Study 2

We designed Study 2 to test the psychological mechanism underlying the face effect, anticipated facial feedback (H1), in a lab experiment. We also examined the interactive effect of sensitivity to face and facial expressiveness on compliance in face-to-face interactions to identify two important boundary conditions of the face effect. Since the successful nonverbal communication of emotions is comprised of both “sending” and “receiving” nonverbal messages accurately (Buck et al., 1972), we predicted that the effect of anticipated facial feedback on compliance would disappear when individuals are insensitive to face, even if the requester’s face is expressive.

### Design, Participants, and Procedure

One hundred and twenty-one students from a North American University were recruited to attend the experiment in exchange for cash payment. The study employed a 2 (sensitivity priming: sensitive to face vs. sensitive to hand) × 2 (facial expressiveness: expressive vs. inexpressive) between-subjects design. Hands were selected in this experiment as a competing channel against face (Hall et al., 2005).

After signing the informed consent forms, participants were first directed to read several pages of step-by-step self-massage instructions and practice the massage skills for several minutes before demonstrating them to the research assistant. The participants were randomly instructed to read an instruction of a facial massage routine to increase their sensitivity to face or an instruction of a hand massage routine to distract attention from face. To verify the success of the manipulation, we performed an analysis of variance (ANOVA) to calculate participants’ rating of their attention to face. The results indicated that participants in the face massage condition rated their sensitivity to face higher than those in the hand massage condition *(M*_face massage_ = 5.72 vs. *M*_hand massage_ = 5.20; *F*(1, 119) = 5.56, *p* = 0.02, and *η^2^* = 0.05).

The research assistant then distributed an ostensibly unrelated questionnaire to participants to collect their responses regarding their willingness to donate half of their participation fee to the local community library. In the expressive condition, the research assistant interacted with participants with lively facial expressions. In the inexpressive condition, the research assistant maintained a relatively impassive facial expression. To avoid suspicion regarding the purpose of the study, we did not include questions related to facial expressiveness in the questionnaire. Rather, we checked the manipulation of facial expressiveness orally during the debriefing. Seventeen participants in the inexpressive condition noted that the research assistant did not demonstrate many facial expressions, and they did not find this inexpressiveness strange.

The dependent measure in our analysis, participants’ willingness to donate, was measured with a Likert-type scale ranging from 1 (very unwilling) to 7 (very willing). As the measurement for anticipated facial feedback, participants indicated their concern for others’ facial feedback using a Likert-type scale ranging from 1 (not at all) to 7 (very much). This is because individuals seeking social approval and taking actions based on other’s facial expressions are concerned about the actual or imagined facial feedback (Modigliani, 1971; Todorov et al., 2008; Ruggiero et al., 2017).

### Results and Discussion

**Figure 2:**
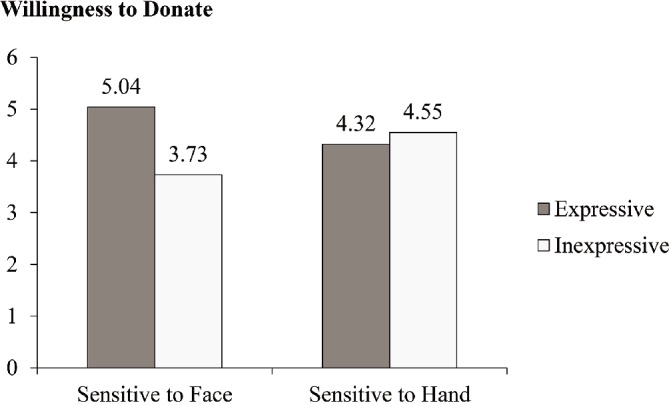
Compliance with donation request as a function of sensitivity priming and facial expressiveness in Study 2.

We performed an ANOVA with sensitivity priming and facial expressiveness as the independent variables and willingness to donate as the dependent variable. The results showed a nonsignificant simple effect of sensitivity priming (*F*(1, 117) = 0.02, *p* = 0.88, and *η^2^* = 0.00) and a nonsignificant simple effect of facial expressiveness (*F*(1, 117) = 2.02, *p* = 0.16, and *η^2^* = 0.02). Consistent with H3, the results revealed a significant two-way interaction between sensitivity priming and facial expressiveness (*F*(1, 117) = 4.09, *p* = 0.05, and *η^2^* = 0.03). As shown in Figure 2, participants who practiced a facial massage on themselves were more willing to donate when the research assistant was expressive relative to when the assistant was inexpressive (*M*_expressive_ = 5.04 vs. *M*_inexpressive_ = 3.73; *t*(117) = 2.44 and *p* = 0.02). In contrast, the willingness to donate the participants who practiced a hand massage on themselves was not significantly affected by the research assistant’s facial expressiveness (*M*_expressive_ = 4.32 vs. *M*_inexpressive_ = 4.55; *t*(117) = −0.43 and *p* = 0.67).

Further, to test whether anticipated facial feedback from the requester drives these effects, we analyzed our data using an SPSS procedure designed by Hayes (2013; PROCESS model 8, sample size = 5,000). We ran a model using participants’ concern for facial feedback as a mediator of the interactive effect of sensitivity priming and facial expressiveness on willingness to donate. The indirect effect of the highest order interaction had a 95% bias-corrected bootstrap confidence interval (CI) ranging from 0.04 to 0.95 (effect size = 0.34 and SE = 0.22), excluding zero. Specifically, the confidence interval ranged from 0.02 to 0.62 (effect size = 0.22 and SE = 0.15) in the face massage (sensitive to face) condition, supporting mediation, while the confidence interval ranged from −0.48 to 0.06 (effect size = −0.12 and SE = 0.13) in the hand massage (sensitive to hand) condition, not supporting mediation. Thus, it provided evidence of a significant indirect effect, supporting H1.

Overall, Study 2 demonstrated that 1) sensitivity to face and requester’s facial expressiveness affect individual compliance in face-to-face interactions and 2) anticipated facial feedback serves as the driving force behind the face effect.

## Study 3

Given that anticipated presence can exert enough social influence and change behavior (Modigliani, 1971), we used videos in Study 3 to simulate face-to-face interactions to test if anticipated facial feedback, not actual facial feedback, was enough to drive the face effect. In this experiment, the participants watched a narrator’s face and its expressions in a video while the narrator was talking with a background voice (except in the written request condition), and the expressiveness of the narrator’s face was varied as a manipulation of the facial feedback that the participants anticipated from the narrator.

We also manipulated inexpressiveness with two different conditions—untimely expression and blank expression—to further test H3 that facial expressiveness of the requester impacts the face effect on individual compliance. The nonverbal communication relies on quick and powerful transmission of the effect (Friedman et al., 1980), suggesting that the immediacy and salience of facial expressions are likely to elicit anticipated facial feedback from the requester in face-to-face interactions. Therefore, we predicted that untimely or blank expressions would lead to inexpressiveness in sending the facial feedback and hence hinder the face effect.

### Participants, Design, and Procedure

Seventy-two students from a North American University participated in this experiment for course credit. After signing the informed consent form, they watched two videos. In the first video, participants watched a short conversation between a narrator, whose face appeared in the center of the camera, and a female background voice. Both male and female narrators were used to rule out any potential effect of gender. The conversation focused on a term paper the narrator was working on. The participants were randomly assigned into one of four groups. For the expressive group, the narrator in the first video displayed appropriate and lively facial expressions when talking to the background voice. For the inexpressive-blank group, the narrator displayed blank facial expressions throughout the video. For the inexpressive-untimely group, the narrator displayed appropriate and lively facial expressions just like in the expressive group. However, each of the facial expressions was delayed for 5–10 s over the conversation. For the written request group, participants answered the compliance question *via* a paper-pencil questionnaire without watching the two videos, hence there was no anticipated facial feedback by video.

Manipulation check showed a significant difference between the three video groups in terms of facial expressiveness (*F*(2, 52) = 7.69, *p* = 0.001, and *η^2^* = 0.23) and timeliness (*F*(2, 52) = 53.41, *p* < 0.001, and *η^2^* = 0.67). Planned contrasts indicated that participants rated facial feedback in the expressive group as significantly more expressive than both the inexpressive-blank group (*M*_expressive_ = 4.33 vs. *M*_inexpressive-blank_ = 2.63; *t*(52) = 3.85 and *p* < 0.001) and the inexpressive-untimely group (*M*_expressive_ = 4.33 vs. *M*_inexpressive-untimely_ = 3.33; *t*(52) = 2.21 and *p* = 0.03). Participants in the inexpressive-untimely group rated the facial expressions significantly less timely than the expressive group (*M*_inexpressive-untimely_ = 1.20 vs. *M*_expressive_ = 4.33; *t*(52) = −10.18 and *p* < 0.001) and inexpressive-blank group (*M*_inexpressive-untimely_ = 1.20 vs. *M*_inexpressive-blank_ = 3.63; *t*(52) = −7.22 and *p* < 0.001). These results suggested that the manipulation of inexpressiveness was successful with both the blank and untimely conditions.

After a filler task, the participants watched the second video, which was the same across all conditions (except the written request group). In the second video, participants again saw the same narrator in the same setting. The narrator asked the participants to choose whether to donate money to a local childcare program. Since the participants were not going to see the narrator after their decision, there would be no actual facial feedback but anticipated facial feedback. We used compliance with the donation request as the dependent variable in this experiment. Participants also rated the attractiveness of the narrator and the weirdness of the recorded video conversation at a seven-point scale as control variables.

### Results and Discussion

**Figure 3:**
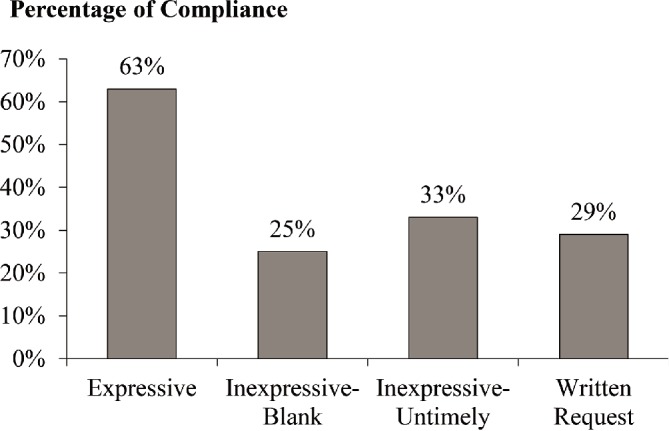
Compliance with donation request as a function of facial expressiveness in Study 3.

The results indicated a significant effect of group on compliance (*χ* ^2^(3) = 7.62 and *p* = 0.06; displayed in Figure 3). A pairwise test showed that the expressive group had a significantly higher percentage of compliance with request than the inexpressive-blank group (63 vs. 25%; *χ* ^2^(1) = 5.07 and *p* = 0.02), the inexpressive-untimely group (63 vs. 33%; *χ* ^2^(1) = 3.03 and *p* = 0.08), and the written request group (63 vs. 29%; *χ* ^2^(1) = 4.17 and *p* = 0.04). There was no significant difference in individual compliance between the inexpressive-blank, inexpressive-untimely, and written request groups (χ^2^(2) = 0.26 and *p* = 0.88).

We also conducted an ANOVA with group as the independent variable and attractiveness of narrator and perceived weirdness of video conversation as dependent variables. The results showed that participants from the three video groups did not perceive the narrator’s attractiveness as significantly different (*F*(2, 52) = 1.47, *p* = 0.24, and *η^2^* = 0.05). Similarly, participants did not perceive the different video conditions as significantly different in terms of weirdness (*F*(2, 52) = 1.32, *p* = 0.28, and *η^2^* = 0.05). These results helped to rule out these two factors as confounding variables.

The results of Study 3 were consistent with findings in Study 1, such that expressive facial feedback can promote compliance with requests relative to the written requests. Further, Study 3 demonstrated that the face effect disappeared when anticipated facial feedback was inexpressive, whether the inexpressiveness was caused by blank or untimely facial expressions. Participants in this study were not going to see the narrator after their decision. Therefore, the results also indicated that the face effect is largely attributable to anticipated, not actual, facial feedback from the requester.

## General Discussion

### Theoretical Contributions

With one field study and two lab experiments, we examine the feedback mechanism that face-to-face interactions generate. Despite the importance of face-to-face interactions in inducing compliance, the mechanism behind the face effect has rarely been explicitly explored (Gerber and Green, 2000; Roghanizad and Bohns, 2017). Taken together, the results show that anticipated facial feedback, not necessarily actual feedback, drives compliance in face-to-face interactions. Specifically, based on research showing that individuals approach positive facial expressions and avoid negative ones (Ruggiero et al., 2017), we show that individuals tend to comply with face-to-face requests due to the concern for the anticipated facial feedback from the requester. The facial feedback, or information conveyed by facial expressions during interpersonal interactions, is an essential factor in social inferences (Todorov et al., 2008; Ueda et al., 2017), and thus is likely to play a critical role in individual compliance. Therefore, this research helps to fill the theoretical void and provides new insights into the psychological process of the face effect.

Moreover, the results show that the face effect is weaker when individuals are not sensitive to face (Studies 1 and 2) or when the requester’s face is inexpressive (Studies 2 and 3). Therefore, we identify two important boundary conditions of the face effect by testing the respective roles of sensitivity to face and requester’s facial expressiveness. In so doing, this research furthers our understanding of factors that affect individual compliance in face-to-face interactions in both the “sending” and “receiving” stages (Buck et al., 1972). Particularly, we showed that inexpressiveness, with either blank faces or untimely facial expressions, mitigates the face effect in the “sending” stage. This increases our understanding from the perspective of requesters (e.g., nonprofit organizations, service staff, and salespeople), which is largely neglected in the social influence research (Bohns, 2016). Therefore, our findings provide important insights into the burgeoning research of individual perceptions of their social influence on others (Roghanizad and Bohns, 2017), as well as present valuable contributions to studies on individual compliance, facial expressions, and communications. With regard to the “receiving” stage, although previous studies have mainly focused on the bright side of interpersonal sensitivity (Kenny et al., 2010; Hall et al., 2015), the current research points to the conditions under which sensitivity backfires. That is, when an individual is sensitive to the face of the interactive partner, she or he is more likely to comply with requests in face-to-face interactions.

Methodologically, we conducted the three experiments with varied individual choices (i.e., donating money, purchasing services and products) in both an Asian city (Study 1) and a North American University (Studies 2 and 3), demonstrating robustness of our findings in different contexts across different cultures. The use of a field experiment, real and video-simulated face-to-face interactions, and differential manipulations of sensitivity to face and facial expressiveness enhances the external validity and generalizability of our findings.

### Empirical Implications

In addition to theoretical contributions, this article also has implications for individuals and practitioners. From the perspective of individuals, this research helps to understand factors that drive and influence compliance tendency in face-to-face interactions. Individuals often face tradeoffs of conflicting interests when asked to comply with a request (Cialdini, 2001). For instance, there is a choice between strengthening a friendship and enjoying personal time when a friend asks an individual to complete a time-consuming task. The mechanism revealed in the present research helps individuals to resist unwanted social influences consciously. This article also indicates how to increase compliance on the requester’s side by, for example, making a request face to face and showing expressive facial expressions. Therefore, this article contributes to individual decisions and welfare improvement.

This research also has considerable implications for practitioners. Consider, for example, that many managers often train salespeople and service staff to simply keep smiling at consumers, showing a relatively inexpressive face. This research indicates that managers could develop more nuanced training related to consumer sensitivity to face and facial expressiveness for salespeople and service staff interacting with consumers face to face. Employees that display demonstrative (both positive and negative, and timely) facial expressions are more likely to gain purchasing compliance from consumers. The face effect also suggests that companies could add face elements to their largely faceless telephone transactions and online sales. With the development of virtual agents, we suggest that the websites must amplify consumer sensitivity to face and provide the virtual agents with expressive and salient human-like faces.

### Limitations and Future Research

This research has several limitations. For instance, it is not clear which type of anticipated facial feedback (i.e., positive or negative) is driving the effects in the present research. Prior work has shown that the effect of negative emotions and feedback such as embarrassment is more pronounced than positive ones (Baumeister et al., 2001; Bohns, 2016). On the other hand, an imagination of positive experience after decision may also increase the likelihood of compliance (Petrova and Cialdini, 2008). Further, it is not clear which specific anticipated feedback (e.g., disgust, anger, sadness, or happiness) is the most effective. Since face-to-face interactions are a complex process and contain numerous nonverbal cues (Short et al., 1976; Song et al., 2016), there can be latent variables other than the anticipated or actual facial feedback. Many direct evidence is needed to examine these questions in future studies.

The current research also opens doors to future research opportunities. One research direction could be in the strategic choice of face-to-face interactions: when will individuals try to engage in, or avoid, face-to-face interactions? Future studies could also investigate other boundary conditions of the face effect, such as situational factors. For instance, frequent or intensive contact from the requester may activate suspicion of her or his ulterior motives (Liu et al., 2019), thus weakening the face effect. The structure of the face-to-face interactions, such as a large number of interactive partners (vs. a few) and the presence of other visual stimuli, can also significantly affect how individuals perceive facial feedback and expressiveness.

## Ethics Statement

The studies in the research were approved by the Ethics Review Boards of the University of Toronto and the School of Economics and Management, Tsinghua University.

## Author Contributions

ML designed and conducted the studies. ML and QZ wrote the paper. QZ analyzed the data. YY provided critical revision of the paper.

### Conflict of Interest Statement

The authors declare that the research was conducted in the absence of any commercial or financial relationships that could be construed as a potential conflict of interest.
